# Dynamic fracture of a dissimilar chain

**DOI:** 10.1098/rsta.2019.0103

**Published:** 2019-09-02

**Authors:** N. Gorbushin, G. Mishuris

**Affiliations:** 1 Laboratoire de Physique et Mécanique des Milieux Hétérogénes (PMMH UMR 7636) CNRS, ESPCI Paris, PSL Research University, 10 rue Vauquelin, 75005, Paris, France; 2 Department of Mathematics, Aberystwyth University, Ceredigion SY23 3BZ, Wales, UK

**Keywords:** brittle fracture, discrete dissimilar structure, crack propagation, subsonic and supersonicsteady-state regimes, Wiener–Hopf technique

## Abstract

In this paper, we study the dynamic fracture of a dissimilar chain composed of two different mass-spring chains and connected with other springs. The propagation of the fault (crack) is realized under externally applied moving forces. In comparison with a homogeneous double chain, the considered structure displays some new essential features of steady-state crack propagation. Specifically, the externally applied forces are of a different strength, unlike a static case, and should be appropriately chosen to satisfy the equilibrium of the structure. Moreover, there exists a gap in the range of crack speeds where the steady-state fracture cannot occur. We analyse the admissibility of solutions for different model parameters and crack speeds. We complement analytical findings with numerical simulations to validate our results.

This article is part of the theme issue ‘Modelling of dynamic phenomena and localization in structured media (part 1)’.

## 1. Introduction

One of the most addressed questions in dynamic problems of fracture is the establishment of the limits of the crack propagation. The limiting crack speed in homogeneous solids is predicted to be a fraction of the Rayleigh speed [1], and a series of experiments [2–6] and numerical simulations [7,8] also demonstrate the growth of instabilities when the crack begins to move at high speed. Although there is much variety in scientific works on dynamic crack propagation in homogeneous solids, papers on interfacial cracks in bi-materials are not as numerous. The latter works have revealed some new phenomena and also provide possible tests for existing fracture theories. Fracture plays an important role in studies of earthquakes, or frictional motion in general [9,10], and it is vital to consider bi-materials for these topics.

An experimental investigation of dynamic fracture in bi-materials was carried out by several research groups. Observations achieved by means of a weight tower device and a gas gun for dynamic crack growth are presented in [11], wherein the authors also attempted to develop a fracture condition based on the complex stress intensity factors for the crack speeds within a subsonic range. The experimental observations were supported by a high-order asymptotic analysis [12]. The study of interfacial steady-state crack growth in [13] proposes the use of the stress intensity factor rather than an energy release rate for these problems, as the latter vanishes and may cause unrealistic computational predictions. Contact between the solids behind a steadily growing crack along the interface in a dissimilar solid is taken into account in [14]. This provided a possible explanation of the experimental observations, shown in the same paper, more confidently. A high contrast in the material properties of a dissimilar solid reveals intersonic crack speeds [15] which are predicted to be inadmissable in homogeneous solids.

A closer inspection of the interface between the solids can reveal new discoveries in the fracture process of bi-materials. For that, a discrete representation of the solid is required. The theoretical analysis of this problem requires advanced mathematical techniques and, therefore, numerical simulations are mostly used [16,17]. The theoretical study [18] examines bi-material solids in the framework of lattice structures but it only considers a quasi-static formulation. Steady-state fault propagation in a dissimilar structure is analysed in [19,20], where the technique developed by Slepyan for crack propagation in lattice structures has been used. In his paramount works [21,22], Slepyan developed a method for linking the microscopic discrete models of dynamic fracture and phase transitions to their macroscopic limits. His results showed the instability of the energy release rate behaviour at low crack speeds, which was later studied in [23], to provide a connection with experimental observations [24,25]. Importantly, the method proposed by Slepyan to solve dynamic fracture problems in discrete structures appears to be extremely efficient and applicable to many configurations; for instance, cracks in triangular [23,26,27] and square-cell lattices [28,29], one-dimensional models for pre-stressed structures [30], chains with non-local interactions [31] and beam structures [32,33]. The reader is referred to the text [34], which shows this technique and covers many aspects of the dynamic features of fracture and phase transitions in discrete systems.

In this work, we will study a one-dimensional model of crack propagation in a double chain of two different materials linked together by elastic springs under mode III fracture. The crack occupies a semi-infinite region and is driven by mobile forces located remotely behind a steadily moving crack tip. The analogous problem for two chains of identical material is thoroughly studied in [35]. Two distinct problems appear to be formally mathematically equivalent to that discussed in this paper: mode II loading of a bi-material structural interface with buckling rods and the fracture of dissimilar chains in mode II configuration in [19,20]. Earlier works on fracture and phase transitions in one-dimensional structures [23,36] demonstrate that not every steady-state solution is admissible. Therefore, we perform an admissibility analysis of the obtained solutions over a wide range of crack speeds. Additionally, we derive expressions for the loading parameters which allow further comparisons with the numerical simulations.

## 2. Problem formulation

**Figure 1.**
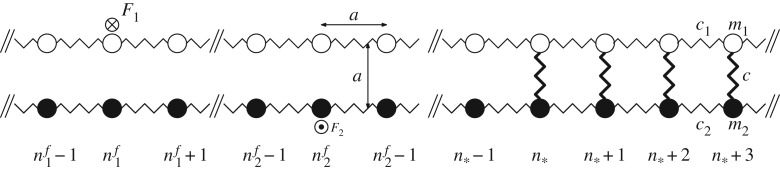
An infinite double chain consisting of dissimilar oscillators linked by massless springs. The stiffnesses of the springs (*c*_1_, *c*_2_) as well as the masses of the oscillators (*m*_1_, *m*_2_) are, in general, different. The two chains are interconnected by linear springs with stiffness *c*, where the intact part of the structure begins with the oscillators of index n_*_, which represents a crack tip moving at the right with some speed *v*.

Let us consider a double chain of dissimilar oscillators with the masses *m*_1_, *m*_2_ of the top and bottom chains, respectively; *c*_1_, *c*_2_—the spring stiffness between them as shown in figure 1. The oscillators are numbered with indices *n*. Different masses with the same index *n*≥n_*_ are connected with each other by linear springs of stiffness *c*. There are external forces *F*_1_, *F*_2_, applied to the top and bottom chains at points *n* = *n*^*f*^_1_ and *n* = *n*^*f*^_2_, respectively.

The equations of motion representing dynamics of a structure are written in the following form:

2.1 $$\begin{array}{rl} m_{1}\frac{d^{2}}{dt^{2}}u_{n}(t) & =c_{1}(u_{n+1}(t)+u_{n-1}(t)-2u_{n}(t))+F_{1}\delta_{nn_{1}^{f}}\\ & \;+c(w_{n}(t)-u_{n}(t))H(n-n_{*})\\ \;\mathrm{and}\;m_{2}\frac{d^{2}}{dt^{2}}w_{n}(t) & =c_{2}(w_{n+1}(t)+w_{n-1}(t)-2w_{n}(t))-F_{2}\delta_{nn_{2}^{f}}\\ & \;-c(w_{n}(t)-u_{n}(t))H(n-n_{*}), \end{array}\}$$

where the displacement *u*_*n*_(*t*) of the *n*th mass of the top chain points towards the plane of the figure, whereas the displacement *w*_*n*_(*t*) of the *n*th mass of the bottom chain points in the opposite direction. *H*(*x*) is the Heaviside step function, i.e. *H*(*x*) = 1, *x*≥0 and *H*(*x*) < 0, *x* < 0. In the following analysis, we assume that the equilibrium separation *a* = 1, which does not influence the results. Moreover, we assume that all quantities with units of length and speed are scaled with *a*.

Masses with index n_*_ = n_*_(*t*) represent a crack tip. The movement of the crack is a result of the breakage of the corresponding link n_*_ = n_*_(*t*) at the concurrent moment *t* = t_*_ such that the following conditions are valid:

2.2 $$|u_{n_{*}}-w_{n_{*}}|=\epsilon_{c},\;|u_{n}(t_{*})-w_{n}(t_{*})|<\epsilon_{c},\;n>n_{*},$$

where *ϵ*_*c*_ = const. is the strength of the springs of stiffness *c*. For the sake of convenience, we introduce the following quantities:

2.3 $$v_{j}=\sqrt{\frac{c_{j}}{m_{j}}}\;\mathrm{and}\;\beta_{j}=\sqrt{\frac{c}{m_{j}}},\;j=1,2.$$

In these notations, *v*_1_ and *v*_2_ are the speeds of sound in the separated top and bottom chains, respectively. We also assume that the applied forces move with constant speeds, *v*^*f*^_*j*_, and that their positions in the structure therefore change according to the following rule:

2.4 $$n_{j}^{f}(t)=v_{j}^{f}t,\;j=1,2,$$

which should be rounded to an integer value. Finally, we introduce the contrasts in the spring constants:

2.5 $$\mu_{j}=\frac{c}{c_{j}},\;j=1,2.$$

This formulation should be supplemented by initial conditions describing the displacements and velocities of each consequent mass along the chain. In the steady-state regime, although the initial conditions play a minor role, their choice remains important for the numerical study of the problem. Here, we try to clarify the significance of *the steady-state regime*. We note that the functions *u*_*n*_(*t*) and *w*_*n*_(*t*)

2.6 $$u_{n}(t)=a_{1}t+a_{0}+n(b_{1}t+b_{0})+u_{n}^{⋆}(t)\;\mathrm{and}\;w_{n}(t)=a_{1}t+a_{0}+n(b_{1}t+b_{0})+w_{n}^{⋆}(t)$$

also represent a solution to the problem if *u*^⋆^_*n*_(*t*) and *w*^⋆^_*n*_(*t*) are found to comprise a limiting (at infinite time) steady-state solution. In the case of a symmetrical chain, the symmetry condition (*u*_*n*_(*t*) = − *w*_*n*_(*t*)) eliminates the possibility of non-uniqueness. However, due to the mismatch in material properties, the constant energy flux delivered by applied (and even symmetrical) forces travels more quickly along the portions of the chains which exhibit higher speeds of sound and will reach the crack tip at an earlier time. This can lead to rigid body motion of the whole system before the solution reaches its possible steady state. Thus, the choice of the originally applied loads, as well as the material properties, may affect the behaviour of the solution in the steady-state regime, as will emerge when analysing the relevant Wiener–Hopf problems. These aspects mainly affect the transient problem and demonstrate the essential dynamic features of such systems.

## 3. Steady-state solution of the problem

The solution to the static problem is discussed in detail in the electronic supplementary material, section S1. In this section, we turn to the dynamic case. Let us assume that, starting from some moment of time, the crack moves with a constant speed *v*. This speed is unknown *a priori* and is a result of the action of two forces *F*_1_ and *F*_2_ moving at speeds *v*^*f*^_1_, *v*^*f*^_2_ < *v*, applied to the chains in a broken portion of the structure (figure 1).

Let us now define the following quantities important for the future analysis:

3.1 $$v_{\max}=\max\{v_{1},v_{2}\},\;v_{c}=\min\{v_{1},v_{2}\}\;\mathrm{and}\;v_{*}=\sqrt{\frac{c_{1}+c_{2}}{m_{1}+m_{2}}},$$

The first parameter defines the limiting value of the crack speed: *v* < *v*_max_. The last, v_*_, defines the speed of the wave propagating along the still intact section of the chain. In the case *v*_1_ = *v*_2_, all three values are equal, *v*_*c*_ = v_*_ = *v*_max_ = *v*_1_, and represent the limiting value for the crack propagation speed in the structure, *v*≤*v*_*c*_. Otherwise, the crack speed can exceed *v*_*c*_. In the general case, *v*_*c*_ < v_*_ < *v*_max_, and we can expect the existence of various propagation regimes depending on the applied load and the material properties of the dissimilar chain. We discuss these cases in detail below, always assuming that *v*_1_≤*v*_2_. In the case of strict inequality, we would have that *v*_*c*_ = *v*_1_, *v*_max_ = *v*_2_ and *v*_1_ < v_*_ < *v*_2_.

Since the crack moves with a constant speed, *v*, it is reasonable to study possible steady-state regimes. Let us express displacements *u*_*n*_(*t*), *w*_*n*_(*t*) as functions of a new variable:

3.2 $$\eta =n-n_{*}(t)=n-n_{0}-vt,$$

where *n*_0_ is the distance between the origin of the laboratory coordinate system and a moving frame at a given time. Following [34], we assume that *η* represents a continuous variable. Consequently, we require that the displacement of the oscillators be expressed as

3.3 $$u_{n}(t)=u(\eta ,t)\;\mathrm{and}\;w_{n}(t)=w(\eta ,t).$$

It is also convenient to consider a linear combination of the displacements:

3.4 ψ(η,t)=u(η,t)−w(η,t)andϕ(η,t)=u(η,t)+w(η,t).

The function *ψ*(*η*, *t*) defines a crack opening at the broken part of the structure, *η* < 0, and is equivalent to an elongation of a spring between the two chains in the intact part of the structure, *η* > 0. The other function *ϕ*(*η*, *t*) describes the change of the central line of the structure over time. The stated fracture criterion (2.2) becomes

3.5 $$\psi (0,t_{*})=\epsilon_{c}\;\mathrm{and}\;|\psi (\eta ,t_{*})|<\epsilon_{c},\;\eta >0.$$

Note that we have eliminated the absolute value requirement here. By adopting the general criterion (2.2)_1_ (or equivalently |*ψ*(0, *t*)| = *ϵ*_*c*_) we may expect that *ψ*(0, *t*) = ± *ϵ*_*c*_ at differing breakage times. This, however, contradicts the assumption of steady-state movement, that the deformation of the structure (at least locally) remains the same when a link breaks. We note that other regular movements are also proved to be possible (see, for example, the alternating regime in [37] or clustering [38] and forerunning [33,39]).

Since the mismatch of the mechanical properties of two chains creates differences in the various possible propagating waves, we should pay attention to respecting the equilibrium condition. We impose the following additional conditions at infinity to avoid possible rigid body motion (compare (2.6)):

3.6 $$\frac{\partial }{\partial t}\phi (\eta ,t)=0,\;t\to \infty$$

and

3.7 |ψ(η,t)|<∞,|ϕ(η,t)|<∞,η>0, t→∞.

We note that the second function, *ϕ*(*η*, *t*), can be determined only up to a constant. In the steady-state regime, all functions become dependent on a single variable *η*:

3.8 $$\{u(\eta ),w(\eta ),\psi (\eta ),\phi (\eta )\}=\lim_{t\to \infty }\{u(\eta ,t),w(\eta ,t),\psi (\eta ,t),\phi (\eta ,t)\}.$$

The fracture criterion reduces to the following in the steady-state regime:

3.9 $$\psi (0)=\epsilon_{c}\;\mathrm{and}\;|\psi (\eta )|<\epsilon_{c},\;\eta >0,$$

and the restrictions (3.7) remain the same.

The steady-state solution to this problem is obtained via application of the Fourier transform

3.10 $$\{U(k),W(k),\Psi (k),\Phi (k)\}=\int_{-\infty }^{\infty }\{u(\eta ),w(\eta ),\psi (\eta ),\phi (\eta )\}\;e^{ik\eta }\;dk,$$

and the Wiener–Hopf technique, which is routinely used for similar problems, as in e.g. [19,22,28,34]. The most important steps in the solution are given in the electronic supplementary material, section S2. Ultimately, the Wiener–Hopf vector problem is reduced to two scalar problems (electronic supplementary material, (S.38) and (S.39)). The essential portion of the solution's derivation relies on the analysis of the characteristic functions

3.11 $$\begin{array}{rl} L(k) & =1+\frac{\beta_{1}^{2}}{{\left(0+ikv\right)}^{2}+\omega_{1}^{2}(k)}+\frac{\beta_{2}^{2}}{{\left(0+ikv\right)}^{2}+\omega_{2}^{2}(k)}\\ \;\mathrm{and}\;M(k) & =\frac{\beta_{1}^{2}}{{\left(0+ikv\right)}^{2}+\omega_{1}^{2}(k)}-\frac{\beta_{2}^{2}}{{\left(0+ikv\right)}^{2}+\omega_{2}^{2}(k)}, \end{array}\}$$

defined by the dispersion relations *ω*^2^_1,2_(*k*) = 4*v*^2^_1,2_sin^2^(*k*/2) on the top and bottom chains, respectively, and (0 ± i*kv*) = lim_*s* → 0+_(*s* ± i*kv*). These functions determine the following important parameters *R* and *Υ*:

3.12 $$R=\exp\left(\frac{1}{\pi }\int_{-\infty }^{\infty }\frac{\mathrm{Arg}\;L(k)}{k}\;dk\right),\;\frac{L(k)}{M(k)}=\Upsilon +O(k^{2}),\;k\to 0,$$

where Arg *L*(*k*) is the complex continuous argument of *L*(*k*). Moreover, the following constant

3.13 $$\Upsilon =\frac{\beta_{1}^{2}(v_{2}^{2}-v^{2})-\beta_{2}^{2}(v_{1}^{2}-v^{2})}{\beta_{1}^{2}(v_{2}^{2}-v^{2})+\beta_{2}^{2}(v_{1}^{2}-v^{2})}$$

depends on the crack speed. The Wiener–Hopf technique allows us to obtain the solution for functions *Ψ*^ ± ^(*k*) (see the electronic supplementary material):

3.14 $$\Psi^{\pm }(k)=\int_{-\infty }^{\infty }\psi (\eta )H(\pm \eta )\;e^{i\;k\eta }\;dk,\;\Psi (k)=\Psi^{+}(k)+\Psi^{-}(k),$$

where the superscripts ‘ ± ’ refer to complex valued functions analytical in the half-planes ± ℑ*k* > 0, respectively. These functions are found to be

3.15 $$\Psi^{+}=\frac{\epsilon_{c}}{0-ik}\frac{1}{L^{+}(k)}\;\mathrm{and}\;\Psi^{-}=\frac{\epsilon_{c}}{0+ik}L^{-}(k),$$

where the functions *L*^ ± ^(*k*) are provided by the factorization of the function *L*(*k*) = *L*^+^(*k*)*L*^−^(*k*), which is inevitably required by this method. The remaining function *Φ*(*k*) is found to be (compare with electronic supplementary material, (S.58)):

3.16 $$\Phi (k)=\epsilon_{c}\left[\frac{\Upsilon }{0+ik}+\left(\Upsilon -\frac{L(k)}{M(k)}\right)\frac{1}{0-ik}\right]L^{-}(k)+C\left(\frac{1}{0+ik}+\frac{1}{0-ik}\right).$$

This form satisfies condition (3.7), as we can estimate from the asymptotic analysis of the integrand. We note here that the arbitrary constant *C* can be fixed, for example, by the restriction *ϕ*(0) = 0 or any other suitable choice.

Finally, the displacements *u*(*η*) and *w*(*η*) can be obtained from a linear combination of the inverse transforms of (3.15) and (3.16).

## 4. Analysis of possible regimes

### (a) Values of applied forces

#### (i) Subsonic regime (*v* < *v*_*c*_)

Within this range of crack speeds, the steady-state regime is achieved under application of the interrelated forces *F*_1_ and *F*_2_:

4.1 $$\frac{F_{j}}{F_{0}}=\frac{\Theta }{R}\frac{v_{j}-v_{j}^{f}}{v_{j}-v},\;j=1,2,\;v<v_{c},$$

where *F*_0_ = *cϵ*_*c*_ is the critical static force needed to break a single spring *c*, extracted from the structure (see electronic supplementary material, section S1). The quantity *Θ* is defined as follows:

4.2 $$\Theta^{2}=\Xi =\frac{\left(\beta_{1}^{2}+\beta_{2}^{2})(v_{*}^{2}-v^{2}\right)}{\left(v_{1}^{2}-v^{2})(v_{2}^{2}-v^{2}\right)},$$

and can be of either sign on the range of crack speed *v* being considered. Note that, in the case where *v*_1_ = *v*_2_ = *v*_*c*_, we recover the condition obtained for the homogeneous chain: *F*_1_/(*v*_*c*_ − *v*^*f*^_1_) = *F*_2_/(*v*_*c*_ − *v*^*f*^_2_) [35]. If the forces move with identical velocities (*v*^*f*^_1_ = *v*^*f*^_2_) then, for the balance condition to be satisfied, we obtain the static balance condition (i.e. the forces are equal). Thus, to preserve the equilibrium of a double chain with a moving crack, the forces applied at different parts of the chain should depend on the achieved crack speed and be generally different.

#### (ii) Intersonic regime (*v*_1_ < *v* < *v*_2_)

In this case, for an evident physical reason, it ensues that the force *F*_1_ does not affect the fracture process, and that only the applied force *F*_2_ affects the possible crack movement. Moreover, there is a gap in the range of crack speeds, *v*_*c*_ < *v* < v_*_, where the crack cannot propagate. We will consider those cases in more detail.

*First intersonic region* (*v*_1_ < *v* < v_*_). No solution exists, with respect to the steady-state ansatz. We note that in this case *Θ*^2^ < 0.

*Second intersonic region* (v_*_ < *v* < *v*_2_). In this case, the energy flux along the chain possessing the speed of sound *v*_1_ does not reach the crack tip, which is travelling at the higher velocity. Hence, it is sufficient to apply the force to another chain given by

4.3 $$\frac{F_{2}}{F_{0}}=\frac{v_{2}-v_{2}^{f}}{R\Xi }\frac{v_{2}+v}{\mu_{2}v_{2}^{2}}\frac{\left(v-v_{1}^{2})(v_{2}^{2}-v_{*}^{2}\right)}{\left(v-v_{*}^{2})(v_{2}^{2}-v_{1}^{2}\right)},\;v_{*}<v<v_{2},$$

where *Ξ* is given in (4.2). The remaining force, *F*_1_, can be taken randomly, which does not influence the steady-state solution up to a constant shift, which depends on the initial location of the forces, and the process preceding the steady movement.

### (b) Displacement fields

**Figure 2.**
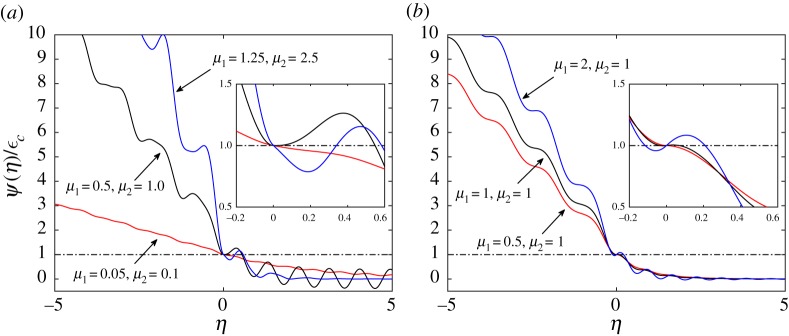
Function *ψ*(*η*) for different values of model parameters: (*a*) *μ*_2_ = 2*μ*_1_, *v*_1_ = *v*_2_, *v* = 0.2*v*_*c*_, (*b*) *μ*_2_ = 1, *v*_1_ = *v*_2_, *v* = 0.3*v*_*c*_. The inserts show magnified plots at the vicinity of a crack tip. (Online version in colour.)

**Figure 3.**
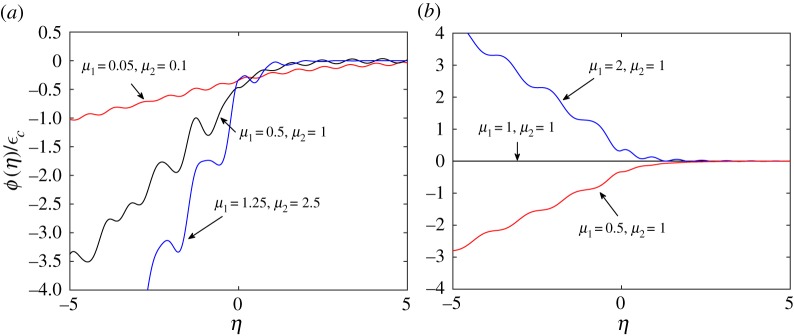
Function *ϕ*(*η*) for different values of model parameters: (*a*) *μ*_2_ = 2*μ*_1_, *v*_1_ = *v*_2_, *v* = 0.2*v*_*c*_, (*b*) *μ*_2_ = 1, *v*_1_ = *v*_2_, *v* = 0.3*v*_*c*_. (Online version in colour.)

We recall that function *ψ*(*η*) expresses the crack opening in the broken part of the structure and the elongation of springs between the chains in the intact part of the double chain. For that reason, it is important to investigate its properties. A plot of *ψ*(*η*) for several cases is shown in figure 2. We also display it together with the results for *ϕ*(*η*) in figure 3, in the same cases.

The feature which is worth of mentioning is that one can see the waves of different lengths emanating from a crack tip both in figures 2 and 3. This peculiarity is common for fracture problems of discrete media and is an essential trait of these problems in comparison with the dynamic fracture of continuum media.

Evaluation of the function *ψ*(*η*) is important for checking the second part of the criterion (3.9), which states the unique location of the crack tip. The violation of such a criterion can be clearly observed in figure 2*a* for *μ*_1_ = 0.5, *μ*_2_ = 1 and *μ*_1_ = 1.25, *μ*_2_ = 2.5, as well as in figure 2*b* for *μ*_1_ = 1, *μ* = 1 and *μ*_1_ = 2, *μ* = 1. The examination of different crack speeds and sets of parameters allow us to distinguish two different sets of solutions. The *admissible* solutions completely fulfil criterion (3.9), whereas for *forbidden* solutions this condition is violated.

In most cases, there only exists a wave behind a crack front, but different scenarios can also emerge. To demonstrate this, we have provided animations in the electronic supplementary material. In those videos, markers show the positions of masses in the numerical simulations and the solid lines are trajectories of the masses, as computed from the analytical solutions. The crack tip, originally located outside of the camera view, propagates from left to right, breaking interfacial springs. We show an example of the steady-state regime where only the reflected wave is present when *c*_1_ = 2, *c*_2_ = 1, *m*_1_ = 2, *m*_2_ = 1, *c* = 1 and *v* = 0.35*v*_*c*_. Certain model parameters also allow the observation of a transmitted wave ahead of a crack tip, as is captured in the video where *c*_1_ = 2, *c*_2_ = 1, *m*_1_ = 2, *m*_2_ = 1, *c* = 0.1 and *v* = 0.2*v*_*c*_. Interestingly, at intersonic crack speeds, should they be possible, the reflected wave propagates only along the chain with the greater speed of sound. That latter case is shown in the last video where *c*_1_ = 2, *c*_2_ = 1, *m*_1_ = 3, *m*_2_ = 1, *c* = 1 and *v* = 1.2*v*_*c*_. These three cases represent the typical scenarios for fracture propagation which appear in this model. A further analysis of admissible solutions is presented in relation to the energy release rate and loads in subsequent sections.

### (c) Energy versus crack speed diagram

**Figure 4.**
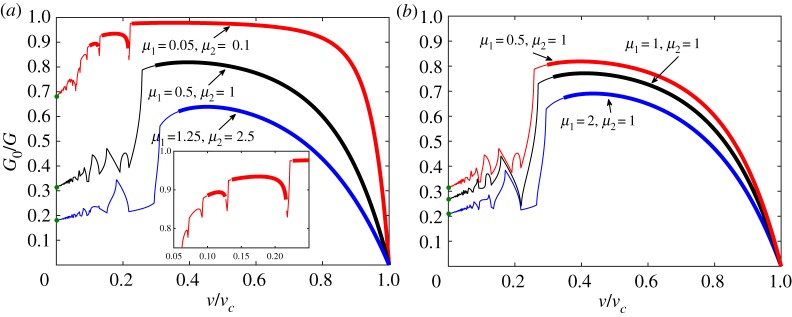
The energy release rates ratio *G*_0_/*G* for different sets of parameters under the condition *v*_1_ = *v*_2_: (*a*) *μ*_2_ = 2*μ*_1_, (*b*) *μ*_2_ = 1. Admissible regimes denoted by thick lines, forbidden regimes denoted by normal lines; green markers stand for the limiting values when *v* → 0, as provided by the quasi-static problem (see the electronic supplementary material). (Online version in colour.)

In this section, we explore the effect of model parameters on the energy release rate and admissible regimes in more detail. The energy release ratio in this case is determined by the following relation [34]:

4.4 $$\frac{G_{0}}{G}=R^{2},$$

where the quantity *G*_0_ = *ϵ*^2^_*c*_*c*/2 is the energy released by breaking a single spring *c* extracted from the structure, and function *R* is given in (3.12). This ratio demonstrates the partition of energy in the system into energy carried by the elastic waves, seen in figure 2, and that spent on the fracture. In other words, more energy is contained in elastic waves for smaller values of this ratio. Several examples of these dependencies are displayed in figure 4. For these examples, we set a constraint on the parameters *v*_1_ = *v*_2_, which allows reduction in the choices for material parameters. We have also marked the limiting values of *G*_0_/*G* as *v* → 0, computed by solving the quasi-static problem shown in the electronic supplementary material. Additionally, we indicate the admissible and forbidden regimes in these plots by the thick and thin lines, respectively.

Figure 4*a* refers to the situation where the stiffness of the vertical springs *c* was varied. An interesting point is that the range of admissible regimes grows with the decrease of *c*. For instance, in the case *μ*_1_ = 0.05, *μ*_2_ = 0.1, there are three distinct intervals of admissible and forbidden regimes. A similar variation in the regimes is also observed for a high contrast in model parameters in a simple chain and a square-cell lattice.

The other plot, figure 4*b*, illustrates the fact that even though the macroscopic properties, such as speeds of sound *v*_1,2_ and stiffness *c* of the springs between the chains, remain the same, the microlevel fracture properties vary. The energy release rates take different values and, moreover, qualitative changes in the admissible regimes occur. We see a growth in the admissible regime with an increase in *μ*_1_ while *μ*_2_ remains constant, for all presented cases. Furthermore, for a chosen crack speed, there are quantitative changes that are reflected in figure 2*b* for *ψ*(*η*), where we see differences in the values of this function and in the wavelengths of the radiated waves.

**Figure 5.**
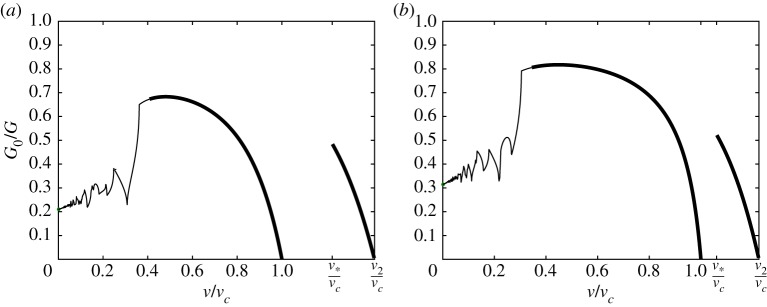
The energy release rates ratio *G*_0_/*G* for different sets of parameters: (*a*) *μ*_1_ = 2, *μ*_2_ = 1, *v*^2^_1_/*v*^2^_2_ = 0.5, (*b*) *μ*_1_ = 0.5, *μ*_2_ = 1, *v*^2^_1_/*v*^2^_2_ = 2/3. Admissible regimes denoted by thick lines, forbidden regimes denoted by normal lines; green markers stand for the limiting values when *v* → 0, as provided by the quasi-static problem (see electronic supplementary material).

In the previous plots, we have studied the cases where *v*_1_ = *v*_2_. The situation differs when these parameters are not equal. In figure 5, we display such examples, where the speeds of sound are different in the two chains. In figure 5*a*, the parameters are chosen in such a way that *v*^2^_1_ = 0.5*v*^2^_2_, whereas in figure 5*b*, we have *v*^2^_1_ = 2/3*v*^2^_2_. Here *v*_2_ is the same in both cases, but according to (2.3) we have different values for *v*_*c*_.

In the presented results, it is interesting to notice that the ratio *G*_0_/*G* takes different values. We can also observe that for smaller *v*_1_ in figure 5*a*, we can achieve lower values of *G*_0_/*G*, in comparison with the results in figure 5*b*. The focus in these plots is attracted by the intervals of *v* that correspond to values *v* > *v*_*c*_. There is a restriction on the range of possible crack speeds within the values $\min\left(v_{1},v_{2}\right),v_{*}$ and $\max\left(v_{1},v_{2}\right)$.

In [20], simplified admissibility conditions were proposed, which reduce to the verification of the following: *u*′(0) < 0. This condition works well for intermediate and high crack speeds but fails to identify the admissible steady states at low speeds, which has been discussed in [35]. Hence, more accurate predictions should be based on the analysis of the complete solution, and not only its derivative as was originally proposed in [23].

Although all the demonstrated results for *G*_0_/*G* reveal interesting features of fracture in the considered structure, we would also like to investigate the dependencies of the force. One minor drawback of *G*_0_/*G* plots is that they are not monotonic even over the intervals of admissible regimes. This, in turn, leads to non-uniqueness in determination of an achieved steady-state crack speed which is supported by different studies [23,26,28,30,34]. Moreover, derived relations for *F*_1_ and *F*_2_ in (2.4) allow easier verification of the determined solution by numerical simulations. Finally, it becomes possible to show the effects of different model parameters on the admissible regimes in terms of the applied load.

### (d) Force versus crack speed diagram

In this section, we analyse the results obtained for the relationships between the forces in (2.4). We have also performed numerical simulations by solving equations (2.1) for a finite number of oscillators with free end boundary conditions. We used 3000 masses of each variety, giving a total of 6000 masses. The forces are located 10 masses away from the original crack tip in order to allow the steady state to be reached more quickly. In the computations, we applied forces calculated for a certain crack speed by (2.4) and recorded the instantaneous crack speed as a function of the fracture time. The instantaneous crack speed eventually stabilized and oscillated around a certain value which, by averaging the corresponding data points, was taken to be a steady-state crack speed. We allowed 1000 fracture events to occur in this process. The settings for these simulations are similar to those in [31], where the details are discussed. We may thus check the validity of (4.1) and (4.3).

All the presented results are given in the case of fixed forces (*v*^*f*^_1_ = *v*^*f*^_2_ = 0). The effects of different values of force speed are shown in [31]. Firstly, we begin with presentations of the results for the cases where *v*_1_ = *v*_2_. From formula (4.1), it follows that in this case:

4.5 $$F_{1}=F_{2}=F.$$

**Figure 6.**
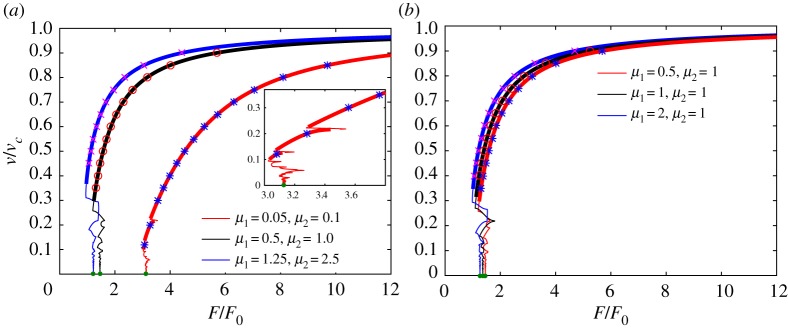
The dependence of normalized force *F*/*F*_0_, according to (2.4) and (4.5), for different sets of parameters under the condition *v*_1_ = *v*_2_: (*a*) *μ*_2_ = 2*μ*_1_, (*b*) *μ*_2_ = 1. Admissible regimes denoted by thick lines, forbidden regimes denoted by normal lines, green markers stand for the limiting values when *v* → 0, as provided by the quasi-static problem (see the electronic supplementary material). The other markers show the results of numerical simulation after solving the dynamical system of equations (2.1). (Online version in colour.)

The dependencies of the force ratio *F*/*F*_0_ are plotted in figure 6. These plots complement those for the energy release ratio in figure 4. The limiting values for *v* → 0 are obtained by solving the corresponding quasi-static problem discussed in the electronic supplementary material. The other markers show results from numerical simulations of (2.1) as described above.

The important feature of the plots of *F*/*F*_0_ is that they provide monotonic correlation between *v* and *F* within the admissible regimes, with the exception of one case, where *μ*_1_ = 1.25, *μ*_2_ = 2.5 in figure 4*a*.

**Figure 7.**
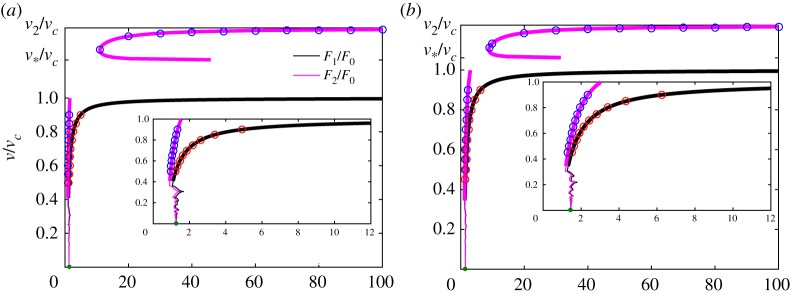
The dependence of the normalized forces *F*_1_/*F*_0_ and *F*_2_/*F*_0_, according to (4.5), for different sets of parameters: (*a*) *μ*_1_ = 2, *μ*_2_ = 1, *v*^2^_1_/*v*^2^_2_ = 0.5, (*b*) *μ*_1_ = 0.5, *μ*_2_ = 1, *v*^2^_1_/*v*^2^_2_ = 2/3. Admissible regimes denoted by thick lines, forbidden regimes denoted by normal lines, green markers stand for the limiting values when *v* → 0, as provided by the quasi-static problem (see the electronic supplementary material). The other markers illustrate the results of numerical simulation after solving the dynamical system of equations (2.1). (Online version in colour.)

An interesting aspect that is not captured by *G*_0_/*G* in the cases of different *v*_1_ and *v*_2_ in figure 5 is that the strengths of the applied forces *F*_1_ and *F*_2_ should be chosen differently to eliminate movement of the entire system as a rigid body. This is shown in figure 7. We note that, if the forces are chosen in a random way, i.e. not belonging to the curves in figure 7, it is still possible to show that there is stable crack propagation, although the displacement would have a linear growth over time (compare (2.6)).

Following the performed numerical simulations, no crack speeds in the range *v*_*c*_ < *v* < v_*_ have been observed. Also, for crack speeds v_*_ < *v* < *v*_2_ (in other words *v*_1_ < *v* < *v*_2_) there was no difference in the obtained results with variation in the force *F*_1_. This confirms that force *F*_2_ plays the major role in fracture when *v*_1_ < *v*_2_, making the other force irrelevant. Indeed, the signal from force *F*_1_, which travels along the chain at a speed lower than the speed of sound *v*_1_ in the upper chain, is not able to reach the crack tip that moves faster than *v*_1_, i.e. *v* > *v*_1_. Interestingly, and this can expected from physical reasons, the system always remains balanced, meaning that it cannot undergo rigid body motion at infinity (*η* > 0).

**Figure 8.**
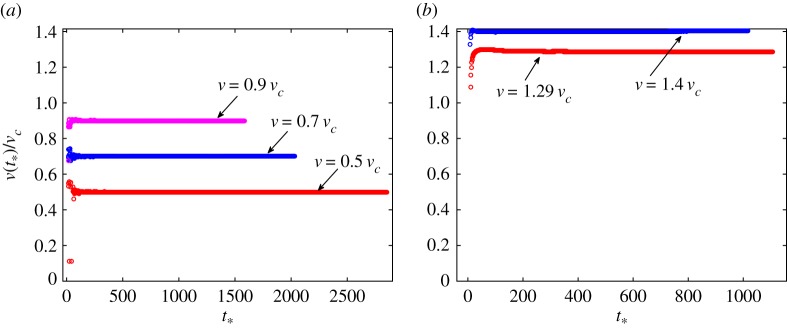
Examples of instantaneous crack speeds for parameter set *μ*_1_ = 2, *μ*_2_ = 1, *v*^2^_1_/*v*^2^_2_ = 0.5: (*a*) subsonic cases, (*b*) intersonic cases. The indicated values *v* correspond to the steady-state crack speeds. (Online version in colour.)

The final remark here concerns the achievement of the steady-state crack speeds. The numerical simulations of the dynamic system of equations allows recording of the instantaneous crack speed. Examples of the data in the case of *μ*_1_ = 2, *μ*_2_ = 1 and *v*^2^_1_/*v*^2^_2_ = 0.5 are shown in figure 8. In that figure, *v*(t_*_) is the crack speed calculated at fracture time t_*_ by means of forward finite differences. The cases of subsonic speeds are shown in figure 8*a*, whereas those of intersonic speeds are displayed in figure 8*b*. As long as the original distance between the crack tip and the forces' location was small enough, the instantaneous crack speed stabilizes relatively quickly to the predicted steady-state value. Notice that oscillations around the established value of the crack speed are very small both in subsonic and intersonic cases.

## 5. Conclusion

It is well known that the fracture process in bi-material solids can reveal effects that are not observed in monomaterials. However, most theoretical works are concerned with continuous media. In this work, we discuss the effects of the mismatches in material parameters that occur in discrete structures. Particularly, we have studied the steady-state separation of a double chain (mode III fracture) by means of moving forces. An analytical solution was derived, as well as expressions that displayed excellent agreement with numerical simulations of the dynamic system.

In this paper (and the electronic supplementary material), we have presented the analytical and numerical analysis of a crack inside the dissimilar chain. The solution demonstrates that the forces must be equal, *F*_1_ = *F*_2_, for the static problem. This scenario changes, however, in the transient regime, where the forces should satisfy certain conditions in order to exhibit the steady-state solution. These conditions encompass the material properties and crack speed, and also connect to the discreteness of the problem.

The solution to the dynamic problem is found by reduction to the vectorial Wiener–Hopf problem which can then be split into two consequential scalar problems. Further analysis of the solution demonstrates that the mismatch in the material properties of the chains leads to essential new peculiarities. Specifically, when the speeds of sound in the chains are different, an intersonic crack speed can occur. Interestingly, there is a gap in the range of possible crack speeds. To be more precise, no steady-state movement can be detected for crack speeds greater than the minimum of the two speeds of sound in the separate chains, and less than the speed of sound in the intact part of the double chain.

Analysis of the analytically computed displacement fields makes it possible to distinguish between the admissible and forbidden steady-state regimes. Forbidden regimes are observed if the fracture condition is met ahead of the principal original crack (where this same condition is always valid). For these regimes, special analysis is required, which lies beyond the scope of the present work. Nevertheless, predictions for the admissible steady-states can be accomplished carefully with the proposed approach and can be confirmed through direct numerical integration of the equations of motion.

Let us recall that similar problems were considered in [19,20] where the dissimilar chains were also compared with the corresponding long wavelength approximation. In the latter, the authors unveiled the possibility of an intersonic fracture propagation and evaluated a simple formula to check admissibility regime border, which we have already mentioned in §4a. We have demonstrated with the use of numerical simulation that admissible regimes in the low-speed domain (if it exists) cannot be predicted by this mentioned formula and need full analysis of the solution. Moreover, we have shown that the evaluation of an exact relationship between the forces allows us to perform the numerical simulations and compare the results with the analytical solution.

The mentioned separation between different steady-state regimes can be conveniently illustrated on the energy release rate or force on the crack speed diagrams. The former allows the estimation of the amount of the energy that is carried by the elastic waves emanating from a crack tip. It can be shown that here, as well as in the quasi-static case, the energy release rate is always higher than the energy released when a single spring is broken. Additionally, it shows the stiffness of the springs connecting the chains plays a significant role in the crack propagation regimes. We have shown that the slow motion of cracks can be observed when the interfacial springs are relatively compliant.

Consideration of the force relationships reveals some intriguing features. The condition imposed on the steady-state system gives two scenarios for the choice of the forces depending on the material properties. If the speeds of sound in two separate chains are the same then the forces should be the same if their location does not change with time. This condition always occurs in the quasi-static formulation of the problem. Distinct speeds of sound in the chain allow the forces to be of different strengths, to avoid rigid body motion of the entire system. Intersonic cracks are possible for identical choices in material properties. Moreover, intersonic crack movement can be caused even by means of one force only, that is applied to the chain with the greater speed of sound.

## Supplementary Material

Supplementary Materials

## Supplementary Material

c1=2,c2=1,m1=2,m2=1,c=0.1,v=0.2vc.mp4

## Supplementary Material

c1=2,c2=1,m1=2,m2=1,c=1,v=0.35vc.mp4

## Supplementary Material

c1=2,c2=1,m1=3,m2=1,c=1,v=1.2vc.mp4

## Data accessibility

This article has no additional data.
