# Supplementary material for: Dynamic fracture of a dissimilar chain
Source: Philos Trans A Math Phys Eng Sci. 2019 Sep 2;377(2156):20190103. doi: 10.1098/rsta.2019.0103 (PMC6732372; doi:10.1098/rsta.2019.0103)
Supplement: Supplementary Materials [file rsta20190103supp1.pdf]

Supplementary Materials. Dynamic fracture of a dissimilar chain.

N.Gorbushin[1] and G. Mishuris[2]

[1] *Laboratoire de Physique et Mécanique des Milieux Hétérogénes (PMMH UMR 7636) CNRS, ESPCI Paris, PSL Research University, 10 rue Vauquelin, 75005 Paris, France*
[2]*Department of Mathematics, Aberystwyth University,Ceredigion SY23 3BZ, Wales, UK*

SM 1 Static problem

The static problem allows derivation of some limiting relationships for a stationary crack when $v \to 0$. We refer to the configuration of a faulted double chain, as displayed in Fig.1, which differs from the Fig. 1 of the main body only with the position of the loads F_1 and F_2, which are applied now at at the left ends of the chains. It is straightforward to conclude that the external forces should be equal, $F_1 = F_2$, to guarantee the equilibrium condition of the structure. This result comes from the solution of the problem which is restricted to the following condition at infinity:

$$u_n, w_n \to 0, \quad n \to +\infty. \tag{S.1}$$

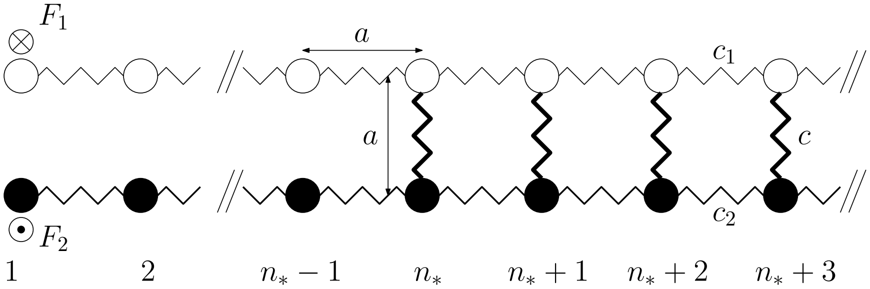

Figure 1: Double chain, where c_1, c_2 are the spring constants of the top and bottom chains, respectively. The two chains are connected together by linear springs of stiffness c, starting from the masses with index n_*, which represents a crack tip.

The solution to the problem can be found once the two parts of the structure, broken and intact, are studied separately. In the intact portion, the equilibrium equations read:

$$\begin{aligned} c_1(u_{n+1} + u_{n-1} - 2u_n) - c(u_n - w_n) = 0, \\ c_2(w_{n+1} + w_{n-1} - 2w_n) + c(u_n - w_n) = 0. \end{aligned} \quad n \geq n_*, \tag{S.2}$$

It is convenient to present the solution in terms of the linear combinations, ψ_n and ϕ_n, of the displacements u_n and w_n:

$$\psi_n = u_n - w_n, \quad \phi_n = u_n + w_n. \tag{S.3}$$

We note that the function ψ_n describes the force in the springs of stiffness c between the corresponding masses for $n \geq n_*$ and the crack opening for $n < n$. The function ψ_n shows the deviation of the middle line of the structure from the symmetry line. As a result, equations (S.2) reduce to the following form:

$$\begin{aligned} \psi_{n+1} + \psi_{n-1} - (2 + \alpha)\psi_n = 0, \\ \phi_{n+1} + \phi_{n-1} - 2\phi_n = \beta\psi_n. \end{aligned} \quad n \geq n_*. \tag{S.4}$$

Here we have introduced the dimensionless parameters:

$$\alpha = \mu_1 + \mu_2, \quad \beta = \mu_1 - \mu_2, \quad \mu_j = \frac{c}{c_j}. \tag{S.5}$$

At the crack tip, $n = n_*$, the fracture condition should also be imposed:

$$\psi_{n_*} = \epsilon_c, \tag{S.6}$$

while at infinity, condition (S.1) leads to:

$$\psi_n, \phi_n \to 0, \quad n \to +\infty. \tag{S.7}$$

The solution of equations (S.4) can be written in the form:

$$\psi_n = \epsilon_c \lambda^{n-n_*}, \quad \phi_n = \epsilon_c \frac{\beta}{\alpha} \lambda^{n-n_*}, \quad n \geq n_*, \tag{S.8}$$

where the factor λ satisfies the condition $|\lambda| < 1$, and should be found from the relevant quadratic equation to be:

$$\lambda = \frac{\sqrt{4+\alpha} - \sqrt{\alpha}}{\sqrt{4+\alpha} + \sqrt{\alpha}}. \tag{S.9}$$

The solution to the broken part of the structure satisfies the system of equations:

$$\begin{gathered} c_1(u_2 - u_1) + F_1 = 0, \quad c_2(w_2 - w_1) - F_2 = 0, \quad n = 1, \\ \left.\begin{aligned} c_1(u_{n+1} + u_{n-1} - 2u_n) = 0, \\ c_1(w_{n+1} + w_{n-1} - 2w_n) = 0. \end{aligned}\right. \quad 1 < n < n_*, \end{gathered} \tag{S.10}$$

and can be found directly as:

$$u_n = \frac{F_1}{c_1}(n_* - n) + u_0, \quad w_n = -\frac{F_2}{c_2}(n_* - n) + w_0, \quad 1 \leq n < n_*,$$

where u_0, w_0 are the displacements at the crack tip. From this we conclude that:

$$\psi_n = \left[\frac{F_1}{c_1} + \frac{F_2}{c_2}\right](n_* - n) + \epsilon_c, \quad \phi_n = \left[\frac{F_1}{c_1} - \frac{F_2}{c_2}\right](n_* - n) + \epsilon_c \frac{\beta}{\alpha}, \quad n < n_* \tag{S.11}$$

Although the formal solution has been derived, we still need to obtain the relationships between the forces that cause the fracture and material properties. For that, we now consider the equations at $n = n_*$, which in terms of ψ_n and ϕ_n are:

$$\begin{gathered} (\psi_{n_*-1} - \psi_{n_*}) + (\psi_{n_*+1} - \psi_{n_*}) - \alpha\psi_{n_*} = 0, \\ (\phi_{n_*-1} - \phi_{n_*}) + (\phi_{n_*+1} - \phi_{n_*}) - \beta\psi_{n_*} = 0 \end{gathered}$$

By utilising the derived solutions in (S.8), (S.11), and the governing equation for λ we reach:

$$\frac{F_1}{c_1} + \frac{F_2}{c_2} = \psi_0 \left(\frac{1}{\lambda} - 1\right), \quad \frac{F_1}{c_1} - \frac{F_2}{c_2} = \psi_0 \frac{\beta}{\alpha} \left(\frac{1}{\lambda} - 1\right).$$

This last set of equations finally allows to determine the forms of the forces:

$$F_1 = F_2 = F, \quad \frac{F}{F_0} = \frac{\sqrt{4+\alpha} + \sqrt{\alpha}}{2\sqrt{\alpha}}, \quad F_0 = c\epsilon_c. \tag{S.12}$$

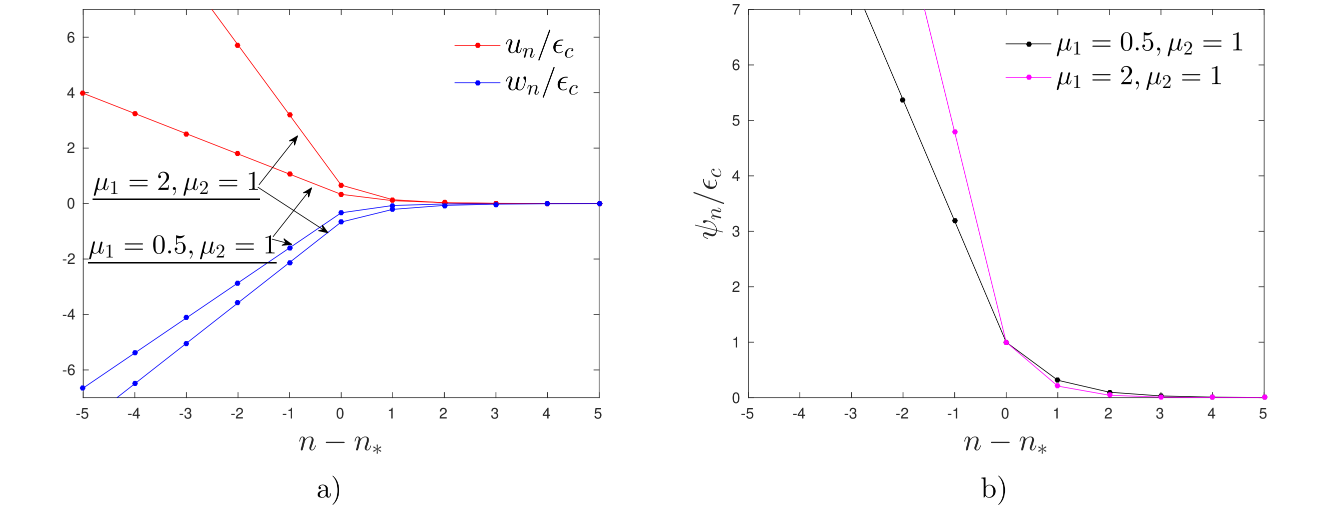

Figure 2: a) The displacements of the chains for two sets of parameters, b) the corresponding function ψ_n for the same sets of parameters.

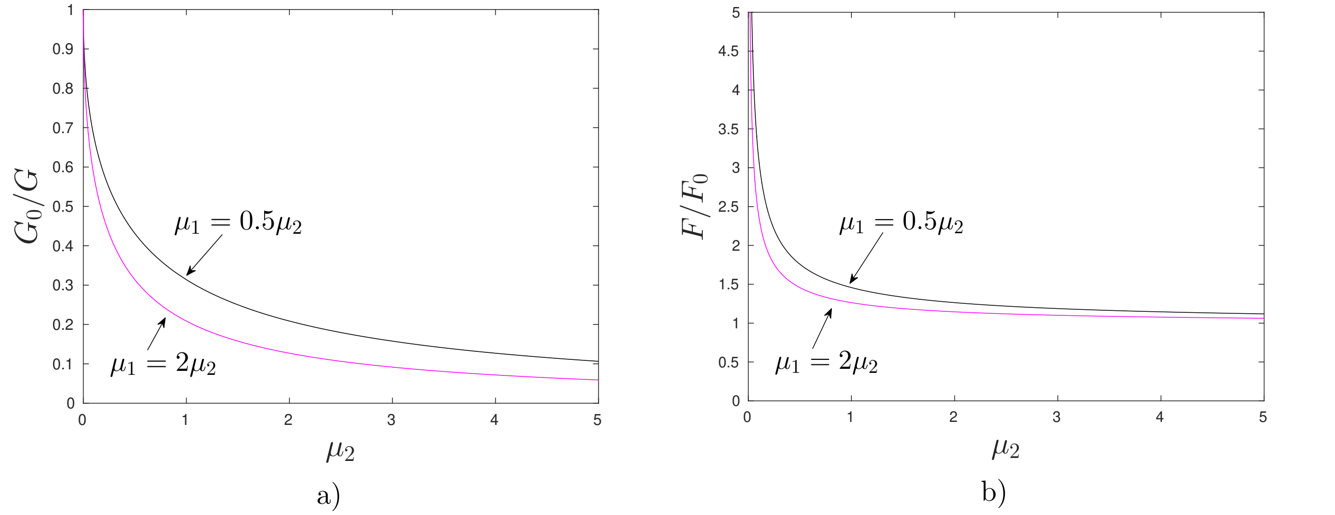

Figure 3: a) The energy release rate ratio G_0/G for two sets of the parameters, b) the force ratio F/F_0 for the same sets of parameters.

Here, F_0 is a static force required to break the structure, composed of two masses connected by a spring of stiffness c. Thus, relations (S.3), (S.8), (S.9), (S.11) and (S.12) solve the problem. The displacements for two particular cases are shown in Fig. 2.

The global energy release rate G can be computed by considering the change in the potential energy of the structure when the crack advances by a unit value length a. This is the same as the difference between the work of forces on the change of displacements and the elastic energies in the broken part of the structure. The energy release rate G is therefore:

$$G = \frac{1}{2a} F_1(u_{n_*-1} - u_{n_*}) - \frac{1}{2a} F_2(w_{n_*-1} - w_{n_*}) = \frac{F_1^2}{2ac_1} + \frac{F_2^2}{2ac_2}$$

Substitution of (S.12) into the last equation results in:

$$G = \frac{G_0}{\alpha}\left(\frac{1}{\lambda} - 1\right)^2, \quad G_0 = \frac{\epsilon_c^2 c}{2a}. \tag{S.13}$$

The quantity G_0 is the local energy release rate, which is equal to the amount of released energy when the structure fails at two masses and a spring of stiffness c within a cell of size a. This quantity complements the force F_0.

The plots for the ratios G_0/G and F/F_0 are presented in Fig. 3 for two sets of material parameters. Some attention should be paid to the fact that for any set of parameters, not only those that were depicted, we observe $G_0/G < 1$ and $F/F_0 > 1$ which is known as the lattice trapping effect [1]. In particular, this effect demonstrates that the force required to break such a discrete structure exceeds the force required to break a single element away from the structure. This is a feature of a discrete model which is not observed in the analysis of continuum solids. It shows that at the microlevel, a special analysis is required.

We would like to highlight that the solution found to the static problem satisfies the fracture condition right at the crack tip and nowhere else. Moreover, in the static case, the total load has to be self-balanced ($F_1 = F_2 = F$) to guarantee that it itself is a trivial conclusion. These two aspects are different in the transient problem, as follows from the main results of the work.

SM 2 Transient problem

SM 2.1 System of Wiener-Hopf equations for a transient regime

For a constant crack speed, the equations of motion in a moving frame $\eta = n - n_*(t)$ become:

$$\begin{gathered}
m_1\left(\frac{\partial^2}{\partial t^2} - 2v\frac{\partial^2}{\partial t\partial\eta} + v^2\frac{\partial^2}{\partial\eta^2}\right)u(\eta,t) = \\
c_1(u(\eta+1,t) + u(\eta-1,t) - 2u(\eta),t) - c(u(\eta,t) - w(\eta,t))H(\eta) + F_1\delta(\eta + n_2 + (v - v_1^f)t), \\
m_2\left(\frac{\partial^2}{\partial t^2} - 2v\frac{\partial^2}{\partial t\partial\eta} + v^2\frac{\partial^2}{\partial\eta^2}\right)w(\eta,t) = \\
c_2(w(\eta+1,t) + w(\eta-1,t) - 2w(\eta),t) + c(u(\eta,t) - w(\eta,t))H(\eta) - F_2\delta(\eta + n_1 + (v - v_2^f)t),
\end{gathered} \tag{S.14}$$

where $H(x)$ is the Heaviside step function, $\delta(x)$ is the Dirac delta-function and $n_j, j = 1,2$ are the distances between the crack tip and top and bottom forces, respectively, at the beginning of crack motion represented by a fixed speed, v.

Consequent applications of the Fourier transform and the Laplace transform (with the terms from the initial conditions being considered irrelevant and omitted in a limiting case $t \to \infty$):

$$\begin{aligned}
\left[(s+ikv)^2 + \omega_1^2(k)\right]U(k,s) &= -\beta_1^2\Psi^+(k,s) + \frac{F_1}{m_1}\frac{e^{-ikn_1}}{s + ik(v - v_1^f)}, \\
\left[(s+ikv)^2 + \omega_2^2(k)\right]W(k,s) &= \beta_2^2\Psi^+(k,s) - \frac{F_2}{m_2}\frac{e^{-ikn_2}}{s + ik(v - v_2^f)},
\end{aligned} \tag{S.15}$$

where

$$\omega_j^2(k) = 4v_j^2\sin^2\left(\frac{k}{2}\right), \quad j = 1,2, \tag{S.16}$$

determine the dispersion relations of the separated chains and are responsible for the wave characteristics of the destroyed part of the structure. Here, the following standard notations have been introduced:

$$\begin{gathered}
U(k,s) = \int_0^\infty\left(\int_{-\infty}^\infty u(\eta,t)e^{ik\eta}\,d\eta\right)e^{-st}\,dt, \quad W(k,s) = \int_0^\infty\left(\int_{-\infty}^\infty w(\eta,t)e^{ik\eta}\,d\eta\right)e^{-st}\,dt, \\
\Psi(k,s) = U(k,s) - W(k,s) = \Psi^+(k,s) + \Psi^-(k,s), \\
\Psi^\pm(k,s) = \int_0^\infty\left(\int_{-\infty}^\infty \psi(\eta,t)H(\pm\eta)e^{ik\eta}\,d\eta\right)e^{-st}\,dt.
\end{gathered} \tag{S.17}$$

The combination of equations (S.15) reduces the problem to a Wiener-Hopf type with respect to the functions $\Psi^{\pm}(k,s)$:

$$\Psi^-(k,s) + L(k,s)\Psi^+(k,s) = \frac{F_1}{im_1(v-v_1^f)}\frac{e^{-ikn_1}}{k-f_1^-}\frac{1}{(s+ikv)^2+\omega_1^2(k)} + \frac{F_2}{im_2(v-v_2^f)}\frac{e^{-ikn_2}}{k-f_2^-}\frac{1}{(s+ikv)^2+\omega_2^2(k)}, \tag{S.18}$$

where

$$f_1^- = \frac{is}{v-v_1^f}, \quad f_2^- = \frac{is}{v-v_2^f}. \tag{S.19}$$

The kernel function $L(k,s)$ is defined as follows:

$$L(k,s) = 1 + \frac{\beta_1^2}{(s+ikv)^2+\omega_1^2(k)} + \frac{\beta_2^2}{(s+ikv)^2+\omega_2^2(k)}. \tag{S.20}$$

We note that function $L(k,s)$ is analytic and not equal to zero as a function of the η-variable along the real axis for any value $s>0$, and the following conditions can be directly checked:

$$L(k,s) = \overline{L(-k,s)}, \quad |L(k,s)| = |L(-k,s)|, \quad \mathrm{Arg}L(k,s) = -\mathrm{Arg}L(-k,s), \quad k\in\mathbb{R}, \quad s\in\mathbb{R}_+,$$
$$L(k,s) = 1 + s^{-2}(\beta_1^2+\beta_2^2) + O(k), \quad k\to 0, \quad L(k,s) = 1 + O\left(\frac{1}{k^2}\right), \quad k\to\infty, \quad s\in\mathbb{R}_+. \tag{S.21}$$

As a result, the index of this function (the winding number) is zero and the function $L(k,s)$ can be factorised for any fixed value of $s>0$ by means of a Cauchy-type integral:

$$L(k,s) = L^+(k,s)L^-(k,s), \quad L^{\pm}(k,s) = \exp\left(\pm\frac{1}{2\pi i}\int_{-\infty}^{\infty}\frac{\mathrm{Log}L(\xi,s)}{\xi-k}\,d\xi\right), \tag{S.22}$$

where the functions $L^{\pm}(k,s)$ are analytic in the half-planes $\pm\Im k>0$, respectively, and both tend to unity as $k\to\infty$. Equation (S.18) can now be rewritten in an alternative manner:

$$\frac{1}{L^-(k,s)}\Psi^-(k,s) + L^+(k,s)\Psi^+(k,s) = B(k,s), \tag{S.23}$$

that makes it directly applicable for application of the Wiener-Hopf technique. We note that the right hand side of the Wiener-Hopf equation

$$B(k,s) \equiv \frac{1}{L^-(k,s)}\sum_{j=1}^{2}\frac{F_j}{im_j(v-v_j^f)}\frac{e^{-ikn_j}}{k-f_j^-}\frac{1}{(s+ikv)^2+\omega_j^2(k)}, \tag{S.24}$$

is a decreasing function along the real η-axis and thus can be presented as a sum of two functions $B^+(k,s)$ and $B^-(k,s)$, which vanish as $k\to\pm\infty$

$$B^{\pm}(k,s) = \frac{1}{2\pi i}\int_{-\infty}^{\infty}B(\xi,s)\frac{d\xi}{\xi-k}, \quad \pm\Im k>0.$$

Then the solution of equation (S.23) takes the form:

$$\Psi^-(k,s) = L^-(k,s)B^-(k,s), \quad \Psi^+(k,s) = \frac{1}{L^+(k,s)}B^+(k,s). \tag{S.25}$$

Function $\Phi(k,s)$ is defined as:

$$\Phi(k,s) = U(k,s) + W(k,s) = \int_0^{\infty}\left(\int_{-\infty}^{\infty}\phi(\eta,t)e^{ik\eta}\,d\eta\right)e^{-st}\,dt, \tag{S.26}$$

and is found from the following equation:

$$\Phi(k,s) = -M(k,s)\Psi^+(k,s) + E(k,s), \tag{S.27}$$

where

$$M(k,s) = \frac{\beta_1^2}{(s+ikv)^2+\omega_1^2(k)} - \frac{\beta_2^2}{(s+ikv)^2+\omega_2^2(k)}. \tag{S.28}$$

and

$$E(k,s) = \frac{F_1}{im_1(v-v_1^f)}\frac{e^{-ikn_1}}{k-f_1^-}\frac{1}{(s+ikv)^2+\omega_1^2} - \frac{F_2}{im_2(v-v_2^f)}\frac{e^{-ikn_2}}{k-f_2^-}\frac{1}{(s+ikv)^2+\omega_2^2}. \tag{S.29}$$

As a result, knowing the function $\Psi^+(k,s)$ from (S.25)$_2$, we can immediately find function $\Phi(k,s)$

$$\Phi(k,s) = -M(k,s)\frac{1}{L^+(k,s)}B^+(k,s) + E(k,s), \tag{S.30}$$

and thus separately determine $U(k,s)$ and $W(k,s)$, which should be inverted to evaluate the sought for displacements $u(\eta,t)$ and $w(\eta,t)$. This is, however, a rather difficult technical task requiring two applications of the Cauchy integral, and then inversion of the Fourier and Laplace integral transforms. On the other hand, the main goal of this research is to analyse possible steady state propagation regimes and determine ways how the transient regimes may approach the former as $t\to\infty$. Here, we may use Abelian type theorems linking the limiting behaviour of functions with their images.

SM 2.2 Wiener-Hopf equations for the steady-state regime

In this section we analyse possible steady-state regimes achieved at $t\to\infty$. In this case, we can reformulate the problem in terms of the Fourier transform only. All the functions become defined by the single variable k:

$$\begin{gathered} U(k) = \lim_{s\to 0+} sU(k,s), \quad W(k) = \lim_{s\to 0+} sW(k,s), \\ \Psi^\pm(k) = \lim_{s\to 0+} s\Psi^\pm(k,s), \quad \Phi^\pm(k) = \lim_{s\to 0+} s\Phi^\pm(k,s), \end{gathered} \tag{S.31}$$

that corresponds to

$$u(\eta) = \lim_{t\to+\infty} u(\eta,t), \quad w(\eta) = \lim_{t\to+\infty} w(\eta,t).$$

The auxiliary functions

$$\psi(\eta) = u(\eta) - w(\eta), \quad \phi(\eta) = u(\eta) + w(\eta), \tag{S.32}$$

satisfy the fracture condition that in the steady-state regime takes the following form:

$$\psi(0) = \epsilon_c, \quad |\psi(\eta)| < \epsilon_c, \quad \eta > 0. \tag{S.33}$$

and condition (3.7) of the main text reads:

$$\int_0^\infty \psi(\xi)d\xi < \infty, \quad \int_0^\infty (\phi(\xi)-\phi_\infty)d\xi < \infty. \tag{S.34}$$

where the integrals should be considered in the sense of distributions while the constant ϕ_∞ is unknown *a priori* and needs to be computed from the solution. This effectively means that the function $\Psi^+(k)$ is bounded near point $k\to 0$, while

$$\Phi^+(k) \sim \frac{\phi_\infty^+}{0-ik}, \quad k\to 0. \tag{S.35}$$

Note also that the function $\phi(\eta)$ (and in some cases also $\psi(\eta)$) can linearly grow as $\eta \to -\infty$. In other words:

$$\Psi^-(k) \sim -\frac{\psi_\infty^-}{(0+ik)^2}, \quad \Phi^-(k) \sim -\frac{\phi_\infty^-}{(0+ik)^2}, \quad k \to 0. \tag{S.36}$$

Finally, since the functions $\phi(\eta)$ and $\psi(\eta)$ are continuous at zero, we can expect that:

$$\Psi^\pm(k) \sim \frac{\psi_0}{0 \mp ik}, \quad \Phi^\pm(k) \sim \frac{\phi_0}{0 \mp ik}, \quad k \to \infty. \tag{S.37}$$

We now multiply equations (S.23) and (S.27) by s and pass it to the limit $s \to 0+$ to transform them to

$$\frac{1}{L^-(k)}\Psi^-(k) + L^+(k)\Psi^+(k) = B(k), \tag{S.38}$$

$$\Phi(k) = -M(k)\Psi^+(k) + E(k), \tag{S.39}$$

where

$$L(k) = \lim_{s\to 0+} L(k,s), \quad M(k) = \lim_{s\to 0+} M(k,s), \quad L^\pm(k) = \lim_{s\to 0+} L^\pm(k,s), \tag{S.40}$$

and

$$B(k) = \lim_{s\to 0+} s\,B(k,s), \quad E(k) = \lim_{s\to 0+} s\,E(k,s).$$

We can check the following estimates:

$$L(k) = \frac{\Xi}{k^2} + O(1), \quad k \to 0, \quad L(k) = 1 + O(k^{-2}), \quad k \to \infty, \tag{S.41}$$

and

$$\frac{M(k)}{L(k)} = \Upsilon + O(k^2), \quad k \to 0, \quad \frac{M(k)}{L(k)} = O(k^{-2}), \quad k \to \infty,$$

where

$$\Xi = \frac{(\beta_1^2+\beta_2^2)(v_*^2-v^2)}{(v_1^2-v^2)(v_2^2-v^2)}, \quad \Upsilon = \frac{\beta_1^2(v_2^2-v^2)-\beta_2^2(v_1^2-v^2)}{\beta_1^2(v_2^2-v^2)+\beta_2^2(v_1^2-v^2)}. \tag{S.42}$$

The behaviour of the factors $L^\pm(k)$ at infinity can be found to be:

$$L^\pm(k) = 1 \pm i\frac{Q}{k} + O\left(\frac{1}{k^2}\right), \quad k \to \infty, \tag{S.43}$$

where the constant $Q = Q(v)$ is computed from the integral:

$$Q = \frac{1}{\pi}\int_0^\infty \log|L(k)|\,dk,$$

which converges due to the estimate (S.41) above.

The asymptotics of the factors $L^\pm(k)$ near the zero point depend essentially on the value of the crack speed v. Depending on the sign of the parameter Ξ from (S.41), we can represent the kernel function $L(k)$ in different manners. Thus if $\Xi = \Theta^2 > 0$, that is true if $v < v_c$ or $v_* < v < v_{max}$, we can write:

$$L(k) = \frac{(\Theta+ik)(\Theta-ik)}{(0+ik)(0-ik)}L_*(k), \quad L^\pm(k) = \frac{(\Theta\mp ik)}{(0\mp ik)}L_*^\pm(k), \tag{S.44}$$

where $L_*(k)$ tends to unity at zero and infinity. As a result, we can prove that

$$L^\pm(k) = \frac{R^{\pm 1}\Theta}{0\mp ik}\left(1 + (0\mp ik)(S+1/\Theta)\right) + O(k), \quad k \to 0, \tag{S.45}$$

where $\Theta = \Theta(v)$, $R = R(v)$ and $S = S(v)$ should be computed from the relationships:

$$R = \exp\left(\frac{1}{\pi}\int_0^\infty \frac{\mathrm{Arg}L_*(k)}{k}\,dk\right), \quad S = \frac{1}{\pi}\int\limits_0^\infty \frac{\log|L_*(k)|}{k^2}\,dk. \tag{S.46}$$

In the remaining case $v_c < v < v_*$, another representation of the kernel $L(k)$ is needed:

$$L(k) = \frac{(\Theta + ik)^2}{(0+ik)^2}L_*(k), \quad L^+(k) = L_*^+(k), \quad L^-(k) = \frac{(\Theta+ik)^2}{(0+ik)^2}L_*^-(k), \tag{S.47}$$

where $\Xi = -\Theta^2 < 0$ in this case. Then the asymptotics of the factors are:

$$L^+(k) = R\big(1 + (0 \mp ik)S\big) + O(k^2), \quad k \to 0, \tag{S.48}$$

$$L^-(k) = \frac{\Theta^2}{R(0+ik)^2}\left(1 + (0+ik)(S + 2/\Theta) + O(k^2)\right), \quad k \to 0, \tag{S.49}$$

where R and S are again computed by the formula (S.46).

We can check that the right hand sides $B(k) \equiv 0$ and $E(k) \equiv 0$ for any $|k| > \varepsilon > 0$. They represent generalised functions (distributions) such that:

$$B(k) = b\left(\frac{1}{0+ik} + \frac{1}{0-ik}\right), \quad E(k) = \frac{e_1}{R\Theta}\left(\frac{1}{0+ik} + \frac{1}{0-ik}\right)L_-(k) - \frac{e_2}{R\Theta}\left(\frac{1}{0+ik} + \frac{1}{0-ik}\right), \tag{S.50}$$

where the coefficients b, e_1 and e_2 take different values depending on the crack speed v and on the loading.

SM 2.3 Solutions of the Wiener-Hopf equations

The solution to the equation (S.38) can be immediately found

$$\Psi^+(k) = \frac{b}{0-ik}\frac{1}{L^+(k)}, \quad \Psi^-(k) = \frac{b}{0+ik}L^-(k). \tag{S.51}$$

Taking into account the asymptotic behaviour of the factors $L^\pm(k)$ given in (S.43) and (S.45), we can immediately find the asymptotics of the solution at infinity:

$$\Psi^\pm(k) = \pm\frac{ib}{k}\left(1 - \frac{Qi}{k}\right) + O\left(\frac{1}{k^3}\right), \quad k \to \infty, \tag{S.52}$$

and at the zero point:

$$\Psi^-(k) = \frac{b\Theta}{R}\frac{1}{(0+ik)^2}\Big(1 + (0+ik)\big(S + 1/\Theta\big)\Big) + O(1), \quad k \to 0,$$
$$\Psi^+(k) = \frac{1}{\Theta}\frac{b}{R} + O(k), \quad k \to 0. \tag{S.53}$$

The fracture condition (S.33) determines the relationship between the loading and the critical elongation ϵ_c:

$$b(v) = \epsilon_c. \tag{S.54}$$

As a result, the solution for function $\psi(\eta) = u(\eta) + w(\eta)$ is expressed in terms of the inverse Fourier transform:

$$\psi(\eta) = \frac{\epsilon_c}{2\pi}\int_{-\infty}^{\infty}\frac{1}{0-ik}\frac{1}{L^+(k)}\,dk, \quad \eta > 0,$$
$$\psi(\eta) = \frac{\epsilon_c}{2\pi}\int_{-\infty}^{\infty}\frac{1}{0+ik}L^-(k)\,dk, \quad \eta < 0. \tag{S.55}$$

and its asymptotics can be computed:

$$\psi(\eta) = \epsilon_c(1 - Q\eta) + O(\eta^2), \quad \eta \to 0,$$
$$\psi(\eta) = \frac{\epsilon_c}{R}\frac{1}{\Theta}\left(-\eta + \left(S + 1/\Theta\right)\right) + O(1), \quad \eta \to -\infty, \tag{S.56}$$
$$\psi(\eta) = O(1), \quad \eta \to \infty.$$

Let us now determine the other auxiliary function $\phi(\eta)$, representing the sum of the displacement (S.32). Equation (S.27) can be conveniently rewritten using (S.51) and (S.54) in the form:

$$\Phi(k) = -M(k)\frac{\epsilon_c}{0 - ik}\frac{1}{L^+(k)} + \frac{e_1}{0 + ik}\left(\frac{1}{0 + ik} + \frac{1}{0 - ik}\right) - e_2\left(\frac{1}{0 + ik} + \frac{1}{0 - ik}\right), \tag{S.57}$$

or

$$\Phi(k) = -\frac{M(k)}{L(k)}\frac{\epsilon_c}{0 - ik}L^-(k) + \frac{e_1}{0 + ik}\left(\frac{1}{0 + ik} + \frac{1}{0 - ik}\right) - e_2\left(\frac{1}{0 + ik} + \frac{1}{0 - ik}\right). \tag{S.58}$$

We can show that the condition

$$e_1(v) = \frac{\epsilon_c \Upsilon(v)}{\Theta(v)R(v)}, \tag{S.59}$$

guarantees that the plus function $\Phi^+(k)$ is bounded near the zero point while the minus function $\Phi^-(k)$ is singular at the zero point. At this point, we can find the sought for function $\phi(\eta)$ by directly applying the inverse Fourier transform.

References

[1] Robb Thomson, C Hsieh, and V Rana. Lattice trapping of fracture cracks. *Journal of Applied Physics*, 42(8):3154–3160, 1971. doi: https://doi.org/10.1063/1.1660699.
